# Emergency Triage Assessment and Treatment Plus (ETAT+): adapting training to strengthen quality improvement and task-sharing in emergency paediatric care in Sierra Leone

**DOI:** 10.7189/jogh.11.04069

**Published:** 2021-12-18

**Authors:** Christopher Hands, Sandra Hands, Madeleine Verriotis, James Bunn, Emma Bailey, Robert J Samuels, Kadiatu Sankoh, Ayeshatu Mustapha, Bhanu Williams, Sebastian Taylor

**Affiliations:** 1 Royal College of Paediatrics and Child Health, London, UK; 2 Institute of Child Health, London, UK; 3 World Health Organisation, Freetown, Sierra Leone; 4 Ministry of Health and Sanitation, Freetown, Sierra Leone

## Abstract

**Background:**

Over the past 25 years Sierra Leone has made progress in reducing maternal and child mortality, but the burden of preventable paediatric deaths remains high. Further progress towards achieving the Sustainable Development Goals will require greater strengthening of the health care system, including hospital care for perinatal and paediatric conditions. Emergency Triage Assessment and Treatment Plus (ETAT+) may offer a useful tool.

**Methods:**

The five-day ETAT+ course was adapted as a six-month programme of in-situ training and mentoring integrated with patient flow and service delivery improvements in 14 regional and district government hospitals across the country. Nurses were trained to carry out the initial resuscitation and assessment of the sick paediatric patient, and to administer the first dose of medication per protocol. The course was for all clinical staff; most participants were nurses.

**Results:**

The intervention was associated with an improvement in the quality of paediatric care and a reduction in mortality. In 2017 mortality decreased by 33.1%, from 14.5% at baseline to 9.7% after six months of the intervention. Mortality at the start of the 2018 intervention was 8.5% and reduced over six months to 6.5%. Care quality indicators showed improvement across the two intervention periods, with some evidence of sustained effect.

**Conclusions:**

These results suggest that adapted ETAT+ training with in-situ mentoring alongside improved patient flow and service delivery supports improvements in the quality of paediatric care in Sierra Leonean hospitals. ETAT+ may provide an affordable framework for improving the quality of secondary paediatric care in Sierra Leone and a model of nurse-led resuscitation may allow for prompt and timely emergency paediatric care in Sierra Leonean hospitals where there are fewer physicians and other resources for care.

Over the last 25 years, Sierra Leone recorded some of the highest maternal, newborn and under-five mortality rates in the world. In 2018 the under-five mortality rate was 105 per 1000 live births, and the maternal mortality ratio was 717 per 100 000 [1]. However, this is neither a complete view of the country’s efforts, nor its more recent achievements. Progress has been made despite severe environmental, demographic and economic challenges, and long-standing weaknesses in the health system. Following the civil war (1991-2002) which undermined health improvement, Sierra Leone saw a 4.5% year-on-year reduction in under-five mortality between 2000 and 2015 – higher than the regional average for sub-Saharan Africa (4.1%) and West and Central Africa (3.7%), putting Sierra Leone in the top quartile of countries globally for improving under-five survival [2].

Following the 2010 launch of the Free Health Care Initiative (FHCI) by the Government of Sierra Leone (GoSL) for children under five, pregnant women, and lactating mothers there was an increase in demand for facility-based care [3]. Between 2008 and 2013, parental reports of care-seeking for children with acute respiratory infections rose from 46% to 72% and with fever from 44% to 66% [4,5]. Developments in roads, communication and ambulance services, as well as urbanisation, are also increasing demand for paediatric hospital services. The capacity of hospitals to respond to this increase in demand is critical to a continued reduction in preventable child mortality. There is a clear need for cost-effective interventions that can improve the quality of care for children presenting to hospitals in Sierra Leone [6].

The GoSL remains heavily dependent on external aid, and aid flows remain volatile inhibiting the ability of the Ministry of Health and Sanitation (MoHS) to plan and prioritise coherent sector development [7-9]. Notwithstanding the major achievement of the FHCI, absolute levels of domestic investment in Sierra Leone’s public hospital network have been weak. Between 2000 and 2015, just 1.02% of health aid to Sierra Leone was earmarked for ‘basic health infrastructure’, and 0.5% to ‘health personnel development’ [10]. Ensuring that secondary facilities in Sierra Leone are equipped and prepared to respond to increasing demand for facility-based care will be critical to sustaining and extending progress on child health and mortality towards achieving the 2030 SDG3 targets [11,12].

The 2014-15 West African Ebola outbreak undermined health care coverage and patient confidence in facility-based care and depleted an already underdeveloped health workforce. Following the outbreak there were 122 doctors and 37 medical specialists for a 7.56 million population, with few working in rural districts [13]. At the start of this programme in 2017, there were 3 physicians per 100 000 people [13]. In the regional hospitals there was one medical officer allocated to cover the paediatrics service; in most district hospitals there was one medical officer to cover the whole hospital. Lack of resources, outdated infrastructure, and low levels of training contributed to poor care and high in-patient paediatric mortality. For Sierra Leone to achieve the 2030 SDG child health goals, it will need an average annual improvement in child mortality of 8%-9.5% [14,15]. This will require significant improvements in health care quality and stronger integration of primary and secondary levels. Inadequate capacity at the secondary level means that hospitals cannot capitalise on gains made in community settings [16].

Short, stand-alone technical training courses often have a limited or poorly sustained impact on clinical skills and quality of care, and indeterminate impact on mortality reduction in low-resource settings [17-19]. Centralised *ex-situ* training courses can be costly and drain scarce clinical personnel from front-line care delivery, achieving results which are unreproducible when participants return to their everyday working environment without substantive change to the systems in which they operate. ETAT+ has been developed as a package of guidelines, training and quality improvement in East Africa, and has become the national framework for facility-based paediatric care in several countries. An elongated *in-situ* model of ETAT+, combining workplace-based training with follow-up practice mentoring, and quality improvement initiatives, may be a more efficient and effective mode of improving service delivery systems in facility-based care. This is the first study to analyse the efficacy of an ETAT+ programme adapted so that initial resuscitation could be delivered by nursing staff where a physician was not available, and the first study to analyse a national ETAT+ programme implemented in West Africa. It is also the first paper to describe an ETAT+ programme delivered over a six-month period alongside in-situ mentorship.

## METHODS

### Intervention

**Figure 1:**
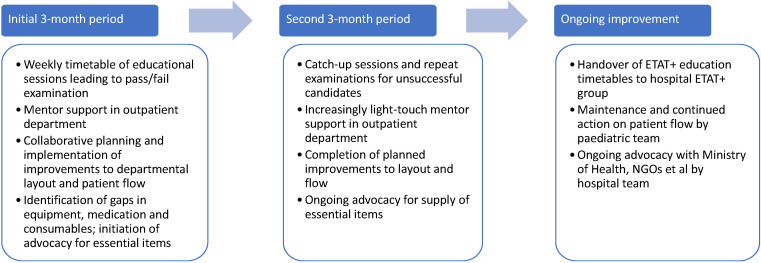
Structure of the adapted ETAT+ programme.

Following a successful pilot in 2015-16 at Ola During Children’s Hospital (ODCH) in Freetown [20], the MoHS incorporated ETAT+ as part of its national child mortality reduction strategy [21]. The five-day ETAT+ course was adapted to be delivered over three months using classroom teaching, skills sessions, simulation and small group discussions, followed by three months’ in-situ practice mentoring with trainees (**Figure 1**).

410 nurses passed the course in 2017 and 317 nurses in 2018. The timetable was constructed around clinical shift patterns and did not interrupt participating nurses’ working day. No incentives were given for attendance at training. Upon completion of the training, the participants undertook written and practical assessments. The written assessment consisted of short answer questions (of which a third of the questions tested prescription chart competencies) and the practical sessions involved simulation of the management of a sick child.

In keeping with the systems approach to quality improvement underpinning ETAT+, a formal assessment of the facility capacity and care bottlenecks was undertaken prior to programme implementation at each hospital. This included an individualised review of patient flow, for example moving the triage point for children close to the paediatric ward, changing the triage process from a number system to an acuity-based system, and establishing a resuscitation area closer to the front gate. The teams also improved access to simple point of care diagnostics (malaria, glucose and haemoglobin); and provided emergency medicines in the resuscitation area where possible, to reduce delays in treatment.

**Figure 2:**
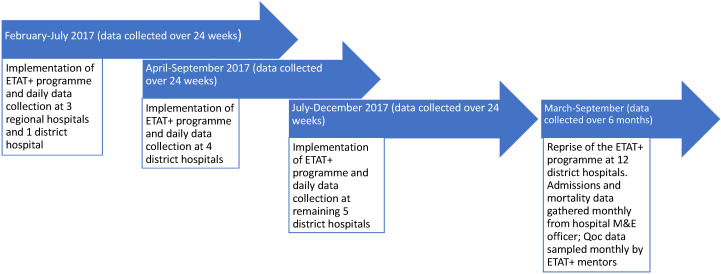
Timetable of implementation and data collection.

With MoHS leadership and support from the UK’s Foreign, Commonwealth, and Development Office (FCDO, formerly DFID) and WHO, a national ETAT+ programme was designed and rolled out by the Royal College of Paediatrics and Child Health to all 13 regional and district government hospitals outside Freetown, the capital city, in three overlapping six-month phases between February and December 2017 [22]. The roll-out included the introduction of national ETAT+ guidelines, and a national triage and stabilisation proforma. A pair of ETAT-trained clinical mentors (a Sierra Leonean nurse from ODCH and an international doctor or nurse) were seconded to each hospital for six months to deliver the adapted three-month ETAT+ course and three months of follow-on clinical support and supervision. After a break of six months, during which mentors were withdrawn, a second iteration of the programme was implemented between March and September 2018 (**Figure 2**).

In 2017, each hospital received its own ETAT mentor team, including one international clinical mentor. In 2018 – reflecting the need to transition to a more sustainable and locally-owned model – international and Sierra Leonean mentor teams were based at four regional ‘hub’ hospitals, providing one-two weeks’ outreach to smaller district facilities each month, working through a locally-established Sierra Leonean clinical ‘ETAT Implementation Group’ in each hospital. In these hospitals staff who had been trained on ETAT+ were frequently moved out of the paediatric ward to other wards, continuously diluting the intervention over time.

The programme mentor teams worked with doctors, nurses and community health officers in each hospital – permanently in situ in the first period, then through permanent basis in regional hospitals and regular outreach visits to smaller district hospitals in the second period – to strengthen triage, resuscitation and emergency treatment capability in each facility. This included enhancing knowledge and skills, improving patient flow, addressing (where possible) infrastructure challenges, and raising issues on the availability and accessibility of essential equipment and medicines, noting that the programme worked within available resources and was unable directly to address those wider deficits. Nurses completing and passing the ETAT+ competency assessments were allowed to give the first dose of emergency medication according to the ETAT+ protocol (oxygen, crystalloid, blood, dextrose, diazepam, phenobarbitone, ampicillin, gentamicin). The programme was supervised by the Child Health team within the Reproductive and Child Health (RCH) Directorate, supported by the Chief Nursing and Midwifery Officer and team.

### Outcome measures

To assess the efficacy of the ETAT+ programme, data relating to triage quality, quality of care, and mortality were formally collected at each hospital.

Triage quality was defined as the time taken for children presenting with emergency or priority signs to be assessed and treated after arrival in the facility. Assessment of triage quality was performed by the mentor teams over a week at the beginning and the end of the six-month intervention in 2017, and at the beginning and after three months in 2018. The method for making these observations has been described previously, [20] and involved the mentor team logging the arrival time of each patient, as well as the time their first treatment was administered. Data for each hospital were logged in an Excel spreadsheet by the mentor team and collated by the programme management team in Freetown.

The quality of emergency treatment was assessed using the following metrics: the management of severe respiratory distress, treatment of severe anaemia with blood transfusion, the treatment of a reduced level of consciousness and seizures, and the accuracy of antibiotic prescribing. In 2017, quality of care was recorded for every patient presenting to the hospital, and the mentor team recorded whether they had been involved in the delivery of care. In 2018, ten sets of case notes per month were randomly selected and reviewed for each indicator assessed.

Data were collected on admissions and deaths. In 2017 mentor teams recorded all paediatric admissions in their hospital, together with the outcome (death, or survival to discharge). In 2018 data on admissions and deaths were collected in collaboration with local ETAT Implementing Groups using the hospital’s own mortality records. Where the hospital had a separate neonatal unit, admissions to that unit were not recorded.

### Statistical analysis

Data sets from two hospitals in 2017 and one in 2018 were incomplete, and these hospitals were excluded from analysis. Data from the remaining three regional and eight district facilities were analysed for both time periods. Analysis was performed with SPSS (v27; IBM, Portsmouth, UK). When assumptions of normality were not met (assessed with Shapiro-Wilk test), non-parametric tests were used. All tests were 2-tailed and assessed at *P* < 0.05.

Triage timing data were assessed at the beginning and end of each intervention (four time points across 2017 and 2018) and were analysed with Jonckheere’s test for ordered alternatives. Mortality and quality of care data were collected across 6 months in each year. In 2017 implementation was staggered across hospitals between February and December and data were therefore collated as six four-week blocks. The 2018 data were collected simultaneously at all hospitals between April and September and were collated by calendar month. Analysis of the quality of care and mortality data was performed across two 3-month blocks using 2×2 χ^2^ tests. Mortality outcomes not recorded were analysed as deaths.

Ethical permission to undertake analysis of these operational data was granted by the Sierra Leone Ethics and Scientific Review Committee.

### Role of the funding source

The funder specified the duration of the intervention, but did not influence the design of the intervention, or of this analysis of the operational data.

## RESULTS

### Patients

In 2017 in the 11 participating facilities 6651 patients were admitted during the 11-month intervention period. In 2018 at 12 participating facilities 14 060 paediatric admissions were recorded over 6 months. Mean admission rates were higher at the three regional hospitals (184 per hospital per month in 2017 rising to 385 per hospital per month in 2018, compared with 70 admissions per month in the district facilities in 2017 rising to 132 in 2018) (Tables S4 and S5 in the **Online Supplementary Documents**).

### Time from triage assessment to treatment

**Figure 3:**
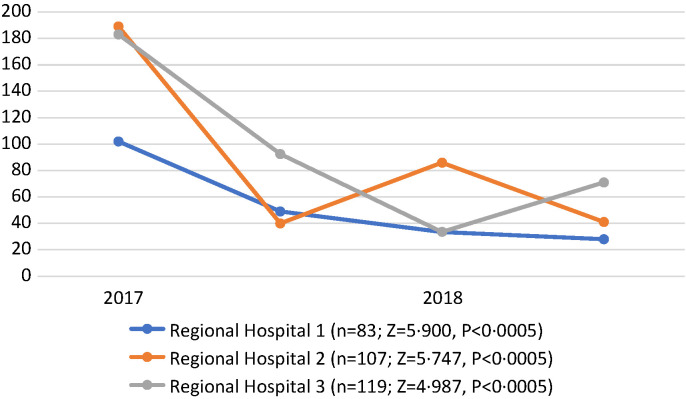
Time from arrival to treatment, 2017-2018 (regional hospitals).

In 2017 there was a reduction over six months in the median time from arrival to first treatment for patients with emergency and priority signs. These timings had not returned to baseline in 2018, and in two out of three centres there was a further reduction (**Figure 3**). The overall reductions in time taken from arrival to first treatment were significant in each hospital across the four measurements: Bo *Z* = 5.900, *P* < 0.0005; Makeni *Z* = 5.747, *P* < 0.0005; Kenema *Z* = 4.987, *P* < 0.0005 (Table S1 in the **Online Supplementary Documents**).

### Quality of care

**Figure 4:**
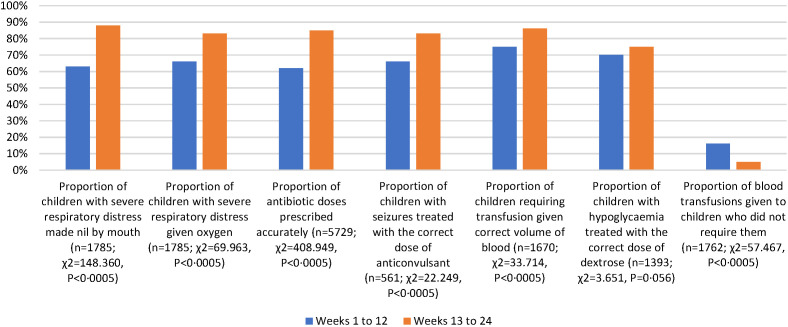
All-hospital quality of care indicators, 2017.

**Figure 5:**
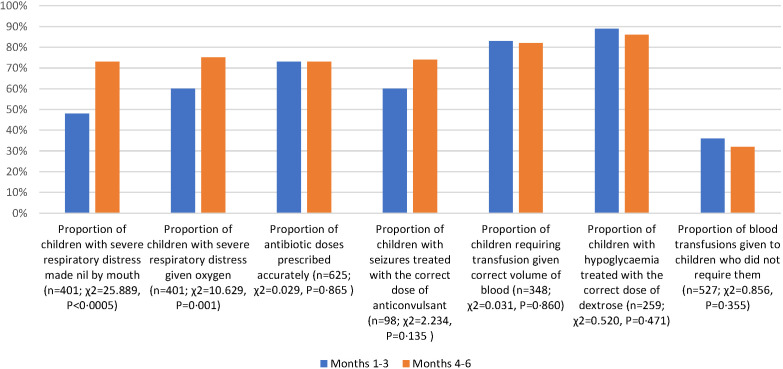
All-hospital quality of care indicators, 2018.

In 2017 all-hospital analysis showed that adherence to guidelines was poor at baseline (Table S2 in the **Online Supplementary Documents**), and that all indicators (other than the treatment of hypoglycaemia) demonstrated significant improvements between the first and second halves of the intervention period (**Figure 4**). In 2018 some of these improvements appear to have been sustained (Table S3 in the **Online Supplementary Documents**), and whilst the quality of care for children with severe respiratory distress improved again, other metrics did not change or changed marginally during the intervention period (**Figure 5**).

### Mortality

**Figure 6:**
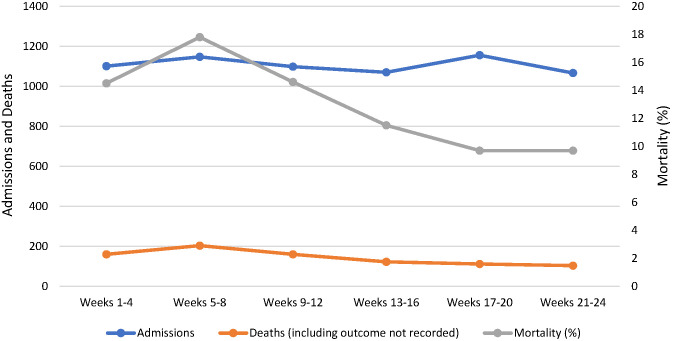
All-hospital paediatric mortality, 2017.

In 2017 paediatric mortality fell from 15.6% in months 1-3 to 10.3% in months four-six (χ^2^ = 42.130, *P* < 0.0005, n = 6639; **Figure 6**). This was despite the proportion of cases in which the ETAT+ mentor pair played an active role in the management of the patient decreasing from 38% in month 1 to 10% in month 6.

**Figure 7:**
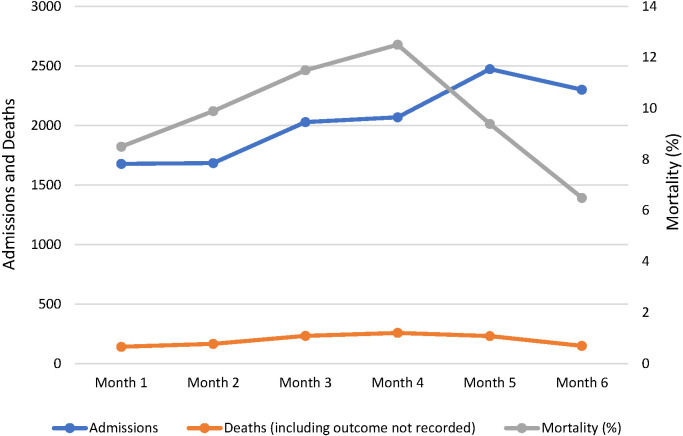
All-hospital paediatric mortality, 2018.

Mortality fell again across participating hospitals in 2018 but not at a level reaching statistical significance (10.1% to 9.4%; χ^2^ = 1.699, *P* = 0.192, n = 12234; **Figure 7**).

**Figure 8:**
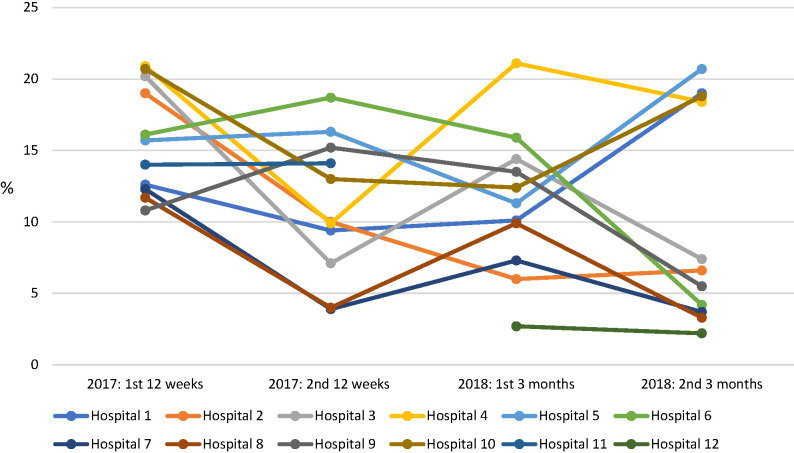
Individual hospital paediatric mortality trends in 2017 and 2018.

There was substantial variation in mortality change across the intervention period among participating hospitals. In 2017, mortality reduction was greater in the regional hospitals. In both periods of intervention, mortality in some district hospitals increased (Table S6 in the **Online Supplementary Documents**). The mortality trends for individual hospitals are presented in **Figure 8**.

## DISCUSSION

This retrospective analysis of programme data shows that a quality improvement approach to paediatric emergency care based on ETAT+ was associated with improvement in markers of care process and quality, and reductions in paediatric mortality in Sierra Leonean government hospitals. These findings are similar to those of studies which have assessed the impact of ETAT+ programmes in other countries [23-25].

This programme differs from those described in earlier papers, because in the Sierra Leonean facilities there were few doctors to cover the hospital. Most members of staff attending the training and leading the initial assessment and resuscitation of sick children were nurses. A task-sharing approach has previously been demonstrated to have benefits in anaesthesia [26] and surgery [27] in Sierra Leone, and these results suggest that a task-sharing approach to paediatric emergency care may also have benefits in Sierra Leone, where district hospitals often have few medical officers.

The 2017 reductions in time from arrival to treatment at regional hospitals suggest that meaningful improvements were made to clinical process and patient flow. Previous studies have suggested a link between improvement in process and speed of access to treatment for the sickest patients, and it is possible that a similar effect is observed here [28,29]. There were further improvements in timing over three months in 2018 despite no further changes being made to layout or flow. This pattern is similar to that observed in the Freetown pilot data and may relate to ongoing improvements in teamwork [20].

The 2017 improvements in quality of care appear to have been sustained across several categories, particularly those relevant to prescribing. However, the management of patients with severe respiratory distress had deteriorated substantially before the 2018 intervention but was amenable to being further improved. Whilst the retrenchment could be due to staff rotation, other factors such as deficits in commodity supply chain or infrastructure may be responsible, particularly as the care of these patients depends on access to a power supply and functioning oxygen concentrator. The interruption of intervention between 2017 and 2018 did not, however, result in reversion to pre-intervention levels of quality of care, indicating some sustained effect. We also see that smaller improvements (or retrenchments) in quality of care indicators in 2018 may reflect a compression effect of smaller available gains, or the effect of other barriers to further improvement. This is helpful in supporting analysis of where and how future interventions should be designed and structured, focusing on underlying systemic blockages to quality, to enhance long-run impact.

The decline in mortality by 34% in 2017 is comparable with reports of reductions in mortality associated with similar interventions in low-resource settings [23,28,29]. Variation of the trend in progress at different hospitals in both 2017 and 2018 suggests the existence of factors which were not (or less) amenable to intervention effect – for example, infrastructural conditions of access to consistent power and clean water, and wider procurement challenges in securing and maintaining vital equipment such as oxygen concentrators or essential medications. Through informal feedback the effect of facility administration and leadership was reported as highly influential in underpinning delivery and impact of ETAT+ intervention. Fostering leadership bridging clinical and administrative staff is a vital ingredient in lasting systemic change in quality of care [30]. There does not appear to have been a rebound in the mortality rate between 2017 and 2018, despite the deterioration in compliance with some clinical protocols shown in **Figure 5**, although this is difficult to interpret given the change in method of recording of admissions and mortality, and the increase in the number of outcomes which were not recorded.

The roll-out of ETAT+ as a national protocol with simultaneous implementation across all regional and district government hospitals may have generated broader systemic benefits beyond individual facilities. By focusing on the most important priorities in the delivery of emergency care for acutely unwell children, we observed that ETAT+ facilitated improved communication between paediatric staff and hospital management, as well as between hospitals and the MoHS, highlighting gaps in the supply chain for medicines and consumables, equipment maintenance and workforce planning. We also see a sizeable increase in demand for facility-based care over 2017-18, possibly at least in part an effect of improving confidence in a system offering higher-quality service, although part of the increase in 2018 may have been due to different methods for defining admissions in some hospitals.

### Limitations

Results reported in this paper are based on operational data captured through programme implementation over two periods. Due to programme funding and design, we were not able to collect data prior to implementation in 2017 so baseline data were collected in the first month of intervention. Similarly, funding was not available for collection of data at non-intervention sites, so a comparison with control sites was not possible. We were not able to isolate and assess quantitatively the influence of ‘external’ factors (workforce changes, water and power supplies, supply chain for equipment and medicines, NGO presence/additional support) on facility processes and outcomes.

Quality of care data were gathered at each site by the programme team, which may have introduced bias, as they were both intervening and collecting data, particularly in 2017 when permanently based in all hospitals, and staff were mentored while providing clinical care, although mentors documented their involvement whenever they assisted in the management of a case. In 2018 the mode of data gathering changed from evaluation of all paediatric admissions by embedded mentor teams to register review for admissions and mortality, and monthly review of a selection of cases by visiting mentor teams to assess quality of care indicators. We recognise that this shift from direct to indirect data collection may have exacerbated vulnerability to errors or omissions, but it also highlights an important trade-off between direct data acquisition in this kind of intervention, and support for strengthening more sustainable local systems. One hospital provided data in 2017 but not in 2018, and another provided data in 2018 but not 2017; this has influenced the periodised mortality rates and potentially comparisons between these periods. The mentor teams at the sites providing incomplete data were unable to gather a full data set due to logistical issues: predominantly the mentors found themselves overwhelmed by clinical need and were not able to give priority to data collection. In this context, the overall trends in process measures and outcomes may be as useful as the comparisons of discrete periods and associated tests of statistical significance.

## CONCLUSION

Strengthening health systems in post-conflict and fragile contexts is a significant challenge. These results suggest that significant improvement in facility-based paediatric care quality is possible under current conditions, with limited additional resource input. This is an important finding in that it works within current structures and staff to deliver the reported improvements in outcomes, and therefore offers good value for money. Further work is needed to assess how best to embed these improvement strategies in the hospital systems of Sierra Leone and similar contexts in the most sustainable manner. International mentors were an important component of the programme design, particularly in catalysing change in the early intensive phase of intervention. Their volunteer participation reduced substantially the cost they imposed on the programme. Within programme design, and responding to funding realities, the input of international mentors has been reduced over time, supporting handover of leadership to local mentors developed as part of the programme strategy. International support maybe continued through remote means, as has been shown during the COVID-19 pandemic, although there may be limitations on remote methods for building the depth of partnership and trust necessary for sustained system change in clinical contexts.

## Additional material


Online Supplementary Document
