# Supplementary material for: Emergency Triage Assessment and Treatment Plus (ETAT+): adapting training to strengthen quality improvement and task-sharing in emergency paediatric care in Sierra Leone
Source: J Glob Health. 2021 Dec 18;11:04069. doi: 10.7189/jogh.11.04069 (PMC8684797; doi:10.7189/jogh.11.04069)
Supplement: Online Supplementary Document [file jogh-11-04069-s001.pdf]

Appendix: online supplementary data

Table S1: Time from arrival to treatment, 2017-2018 (regional hospitals)

**Hospital (number of patients)**	**2017 median times from arrival to treatment in mins (IQR)**		**2018 median times from arrival to treatment in mins (IQR)**		**Jonckheere's test (assessment of all 4 observations)**
	Month 1	Month 6	Month 1	Month 3	
**Hospital 1(n=83)**	102 (68-174)	49 (25-68)	33·5 (26-42)	28 (21-42)	z=5·900, p<0·0005
**Hospital 2(n=107)**	189 (121-271)	40 (24-53)	86 (36-107)	41 (32-56)	z=5·747, p<0·0005
**Hospital 3 (n=119)**	183 (126-215)	92·5 (58-109)	33·5 (25-61)	71 (36-108)	z=4·987, p<0·0005

Table S2: All-hospital quality of care indicators, 2017

	Four-week block								
**Indicator**	1	2	3	4	5	6	1-3	4-6	Chi-square (1-3 vs 4-6)
**Percentage of children with severe respiratory distress made nil by mouth (n=1785)**	100/227 (44)	185/277 (67)	229/306 (75)	238/283 (84)	283/318 (89)	336/374 (90)	514/810 (63)	857/975 (88)	148.360, p<0·0005
**Percentage of children with severe respiratory distress given oxygen (n=1785)**	117/227 (52)	202/277 (73)	215/306 (70)	218/283 (77)	244/318 (77)	348/374 (93)	534/810 (66)	810/975 (83)	69.963, p<0·0005
**Percentage of antibiotic doses prescribed accurately (n=5729)**	345/890 (39)	618/955 (65)	744/923 (81)	812/942 (86)	871/1029 (85)	839/990 (85)	1707/2768 (62)	2522/2961 (85)	408.949, p<0·0005
**Percentage of children requiring transfusion given correct volume of blood (n=1670)**	126/226 (56)	208/246 (85)	232/280 (83)	242/278 (87)	298/347 (86)	253/293 (86)	566/752 (75)	793/918 (86)	33.714, p<0·0005
**Percentage of blood transfusions given to children who did not require them (n=1762)**	67/269 (25)	36/269 (13)	30/293 (10)	38/301 (13)	7/346 (2)	2/284 (1)	133/831 (16)	47/931 (5)	57.467, p<0·0005
**Percentage of children with hypoglycaemia treated with the correct dose of dextrose (n=1393)**	101/172 (59)	156/224 (70)	179/224 (80)	204/254 (80)	192/258 (74)	183/261 (70)	436/620 (70)	579/773 (75)	3.651, p=0·056
**Percentage of children with seizures treated with the correct dose of anticonvulsant (n=561)**	45/84 (54)	64/92 (70)	82/113 (73)	96/107 (90)	71/93 (76)	60/72 (83)	191/289 (66)	227/272 (83)	22.249, p<0·0005

Table S3: All-hospital quality of care indicators, 2018

	Month								
**Indicator**	1	2	3	4	5	6	1-3	4-6	Chi-square
**Proportion of children with severe respiratory distress made nil by mouth (n=401)**	26/51 (51%)	33/63 (52%)	34/78 (44%)	41/74 (55%)	53/62 (85%)	59/73 (81%)	93/192 (48%)	153/209 (73%)	25.889, p<0·0005
**Proportion of children with severe respiratory distress given oxygen (n=401)**	27/51 (53%)	34/63 (54%)	54/78 (69%)	46/74 (62%)	52/62 (84%)	59/73 (81%)	115/192 (60%)	157/209 (75%)	10.629, p=0·001
**Proportion of antibiotic doses prescribed accurately (n=625)**	64/105 (61%)	78/101 (77%)	90/110 (82%)	84/105 (80%)	66/98 (67%)	75/106 (71%)	232/316 (73%)	225/309 (73%)	0.029, p=0·865
**Proportion of children requiring transfusion given correct volume of blood (n=348)**	37/46 (80%)	50/57 (88%)	51/63 (81%)	54/68 (79%)	48/54 (89%)	48/60 (80%)	138/166 (83%)	150/182 (82%)	0.031, p=0·860
**Proportion of blood transfusions given to children who did not require them (n=527)**	31/77 (40%)	27/84 (32%)	35/98 (36%)	26/94 (28%)	31/85 (36%)	29/89 (33%)	93/259 (36%)	86/268 (32%)	0.856, p=0·355
**Proportion of children with hypoglycaemia treated with the correct dose of dextrose (n=259)**	42/49 (86%)	41/45 (91%)	43/47 (91%)	33/41 (80%)	38/44 (86%)	31/33 (94%)	126/141 (89%)	102/118 (86%)	0.520, p=0·471
**Proportion of children with seizures treated with the correct dose of anticonvulsant (n=98)**	12/19 (63%)	7/11 (64%)	12/22 (55%)	18/22 (83%)	8/11 (73%)	8/13 (62%)	31/52 (60%)	34/46 (74%)	2.234, p=0·135

Table S4: All-hospital paediatric mortality, 2017

	Admissions	Discharged alive	Deaths	Outcome not recorded	Deaths + outcome not recorded	Mortality (%)	Periodised mortality (%)
**Month 1**	1101	941	139	21	160	14·5	15·7
**Month 2**	1148	944	162	42	204	17·8	
**Month 3**	1099	939	157	3	160	14·6	
**Month 4**	1070	947	123	0	123	11·5	10·3
**Month 5**	1156	1044	101	11	112	9·7	
**Month 6**	1067	963	91	13	104	9·7	

Table S5: All-hospital paediatric mortality, 2018

	Admissions	Discharged alive	Deaths	Outcome not recorded	Deaths + outcome not recorded	Mortality (%)	Periodised mortality (%)
**Month 1**	1678	1536	116	26	142	8·5%	10·1%
**Month 2**	1685	1518	136	31	167	9·9%	
**Month 3**	2028	1795	129	104	233	11·5%	
**Month 4**	2069	1811	124	134	258	12·5%	9·4%
**Month 5**	2474	2242	98	134	232	9·4%	
**Month 6**	2300	2150	108	42	150	6·5%	

Table S6: Individual hospital paediatric mortality trends in 2017 and 2018

		2017				2018
**Hospital**	1st 12 weeks	2nd 12 weeks	Chi-square	1st 3 months	2nd 3 months	Chi-square
1 (R)	64/509 (12·6%)	74/788 (9·4%)	3.295, p=0·069 (n=1297)	117/1159 (10·1%)	233/1227 (19%)	37·670, p<0·0005 (n=2386)
2 (R)	84/442 (19%)	39/389 (10%)	13·227, p<0·0005 (n=831)	64/1059 (6%)	120/1829 (6·6%)	0·301, p=0·583 (n=2888)
3 (R)	108/534 (20·2%)	46/646 (7·1%)	44·238, p<0·0005 (n=1180)	116/806 (14·4%)	63/851 (7·4%)	20·984, p<0·0005 (n=1657)
4 (D)	47/225 (20·9%)	21/212 (9·9%)	10·021, p=0·002 (n=437)	40/190 (21·1%)	45/245 (18·4%)	0·491, p=0·484 (n=435)
5 (D)	61/389 (15·7%)	52/319 (16·3%)	0·050, p=0·823 (n=708)	31/274 (11·3%)	68/329 (20·7%)	9·534, p=0·002 (n=603)
6 (D)	24/149 (16·1%)	20/107 (18·7%)	0·292, p=0·589 (n=256)	21/132 (15·9%)	11/265 (4·2%)	16·438, p<0·0005 (n=397)
7 (D)	22/179 (12·3%)	8/206 (3·9%)	9·421, p=0·002 (n=385)	18/246 (7·3%)	12/326 (3·7%)	3·730, p=0·053 (n=572)
8 (D)	22/188 (11·7%)	5/125 (4%)	5·651, p=0·017 (n=313)	19/191 (9·9%)	7/215 (3·3%)	7·557, p=0·006 (n=406)
9 (D)	56/520 (10·8%)	57/376 (15·2%)	3·816, p=0·051 (n=896)	86/638 (13·5%)	38/695 (5·5%)	25·309, p<0·0005 (n=1333)
10 (D)	19/92 (20·7%)	7/54 (13%)	1·374, p=0·241 (n=146)	14/113 (12·4%)	27/144 (18·8%)	1·910, p=0·167 (n=257)
11 (D)	17/121 (14·0%)	10/71 (14·1%)	0.000, p=0·995 (n=192)			
12 (D)				16/583 (2·7%)	16/717 (2·2%)	0.352, p=0·553 (n=1300)
